# DNA index determination with Automated Cellular Imaging System (ACIS) in Barrett's esophagus: Comparison with CAS 200

**DOI:** 10.1186/1472-6890-5-7

**Published:** 2005-08-12

**Authors:** Qin Huang, Chenggong Yu, Michael Klein, James Fang, Raj K Goyal

**Affiliations:** 1 Center for Swallowing and Motility Disorders, Departments of Medicine & Pathology and Laboratory Medicine, VA Boston Healthcare System and Harvard Medical School, 1400 VFW Parkway, West Roxbury, MA 02132, USA; 2 Department of Pathology and Laboratory Medicine, Providence VA Medical Center and Brown Medical School, Providence, RI 02908, USA

## Abstract

**Background:**

For solid tumors, image cytometry has been shown to be more sensitive for diagnosing DNA content abnormalities (aneuploidy) than flow cytometry. Image cytometry has often been performed using the semi-automated CAS 200 system. Recently, an Automated Cellular Imaging System (ACIS) was introduced to determine DNA content (DNA index), but it has not been validated.

**Methods:**

Using the CAS 200 system and ACIS, we compared the DNA index (DI) obtained from the same archived formalin-fixed and paraffin embedded tissue samples from Barrett's esophagus related lesions, including samples with specialized intestinal metaplasia without dysplasia, low-grade dysplasia, high-grade dysplasia and adenocarcinoma.

**Results:**

Although there was a very good correlation between the DI values determined by ACIS and CAS 200, the former was 25% more sensitive in detecting aneuploidy. ACIS yielded a mean DI value 18% higher than that obtained by CAS 200 (p < 0.001; paired *t *test). In addition, the average time required to perform a DNA ploidy analysis was shorter with the ACIS (30–40 min) than with the CAS 200 (40–70 min). Results obtained by ACIS gave excellent inter-and intra-observer variability (coefficient of correlation >0.9 for both, p < 0.0001).

**Conclusion:**

Compared with the CAS 200, the ACIS is a more sensitive and less time consuming technique for determining DNA ploidy. Results obtained by ACIS are also highly reproducible.

## Background

By molecular pathology, Barrett's adenocarcinoma has been shown to be an acquired malignancy with multi-genetic alterations involving almost all chromosomes [1,2]. These observations have indicated that numerical and structural chromosomal abnormalities may be important events in the carcinogenic process in Barrett's adenocarcinoma. This is especially important inasmuch as there is accumulating evidence that DNA aneuploidy is a key early event in tumorigenesis and may be a cause rather than a consequence of malignancy [3,4]. Chromosomal abnormalities occur predominantly in aneuploid malignant cells, leading to progressive deterioration of aneuploidy [5]. In addition to changes in chromosome copy numbers, cancer cells may also experience a change in the size of individual chromosomes, due to chromosomal translocations, deletions, and duplications. In flow or image cytometry of cancer cells, DNA content is represented by the DNA index (DI) and described in reference to the DI of normal diploid cells. DI values reflect integrated optical density (IOD), which takes into account chromosome copy number, extra chromosomal fragments and the DNA content of individual chromosomes, as well as morphometric features of the nuclei. Thus, DI values reflect more than the copy number of the chromosomes in a nucleus, and the term aneuploidy, which was originally introduced to indicate only changes in chromosome copy number, is now used to indicate any changes in the DI of cancer and pre-cancerous cells.

DNA aneuploidy has been used as a marker of cancers that exhibit more aggressive behavior than diploid cancers, and as a marker of pre-malignant lesions that are at high risk for malignant progression to cancer. Although DNA aneuploidy has usually been measured by flow cytometry, the sensitivity of this method is limited in diagnosing DNA content abnormalities because it includes both affected cells as well as benign epithelial and stromal cells in the test sample. Image cytometry has recently been used to estimate the DI of cells in disaggregated cytospin preparations or in microscopically identified epithelial cells [4]. Image cytometry has been shown to be more sensitive than flow cytometry for analyzing DNA content [6,7]. Image cytometry has been performed using a number of systems, the most common being the semi-automated CAS 200 system [8-10]. Recently, a more automated system, the Automated Cellular Imaging System (ACIS) (ChromaVision Medical System, Inc., San Juan Capistrano, CA) was introduced for the analysis of DNA ploidy, but results using this system have not been validated against the conventional CAS 200 system. Using these two systems, in the present work we compared the DNA index (DI) obtained from the same archived formalin-fixed and paraffin embedded tissue samples from Barrett's esophagus related lesions, including those with dysplasia and adenocarcinoma. We found that the two systems gave correlated DI values and that the ACIS system gave higher DI values, diagnosed aneuploidy more frequently, and may be less labor intensive than the CAS 200 system. Furthermore, results obtained with ACIS were highly reproducible.

## Methods

### Tissue samples

A total of 34 distal esophageal biopsies or distal esophageal resection specimens containing Barrett's esophagus related lesions were collected from 13 patients. The DI of these tissue samples had been examined using the CAS 200 (Becton Dickenson, San Jose, CA) [8] and were subsequently assayed using the ACIS instrument. The study protocol was approved by the Institutional Review Boards of the Boston and Providence Veterans Affairs Medical Centers.

### Histology

The tissue blocks were sectioned to obtain two adjacent sections, one stained with H&E and the other with Feulgen dye. The H&E stained sections were examined by two experienced pathologists (Q.H. and M.K.) for consensus diagnoses of Barrett's esophagus with specialized intestinal metaplasia (SIM), without dysplasia (BE), and for low-grade dysplasia (LGD), high-grade dysplasia (HGD), and esophageal adenocarcinoma (EAC), according to published criteria [11,12]. Briefly, SIM was diagnosed if the esophageal epithelium was columnar and contained characteristic goblet cells [11]. A diagnosis of LGD depended primarily on prominent cytological changes such as enlarged nuclei with hyperchromasia and increased number of mitoses as well as mild architectural changes. The crypt architecture in LGD was slightly distorted but generally preserved. The epithelial nuclei were enlarged, crowded, and hyperchromatic with increased mitotic figures, and the changes were present in the upper portion of the crypt. The nuclei remained polarized but were slightly stratified, and the stratification did not reach the apical surface of the glands. Mucus in the goblet and columnar cells was diminished or absent. In contrast, samples with high-grade dysplasia (HGD) showed marked cytological and architectural changes, including abnormal glandular proliferations with villiform and cribriform growth patterns in the upper portion of the mucosa, and marked distortion of crypt architecture with branching, lateral budding of crypts, and "back-to-back" patterns [12]. Nuclear stratification reached the crypt luminal surface, with loss of nuclear polarity, and nuclei varied markedly in size, shape and staining characteristics. The nuclear abnormalities extended to the mucosal surface. Mucin production in the goblet and columnar cells was usually absent. Esophageal adenocarcinoma (EAC) was diagnosed when dysplastic columnar epithelial cells invaded through the basement membrane into the muscularis mucosa, submucosa, and beyond.

### DNA index (DI) determination by CAS 2000

For analysis on the CAS 200 System, fixed 5 μm tissue sections were Feulgen-stained using the Quantitative DNA Staining Kit (Cell Analysis Systems, Inc., Elmhurst, IL) as recommended by the manufacturer. This staining process involves hydrolysis with concentrated hydrochloric acid, that removes non-nuclear substances and hydrolyses chromatin into its constituent nucleic acids, followed by stoichiometric binding of the dye to the nucleic acids, imparting a blue color. The intensity of the blue color is directly proportional to the amount of DNA. Areas on the Feulgen-stained sections that contained pathological lesions, as defined by adjacent H&E stained slides, were marked for DNA content analysis. The corresponding areas on the Feulgen-stained sections were scanned, digitized, and stored as individual files using the CAS 200 quantitative DNA software program [13]. Artifacts, including overlapping nuclei, were edited out. The digitized images of microscopically selected nuclei were converted into a series of pixels, which were quantified on the basis of parameters such as the integrated optical density (IOD), reflecting the DNA content of the selected nucleus. CAS 200 also uses a tissue correction software, which corrects for underestimation of DI due to cutting off of the nuclei. Both the Feulgen staining and ploidy analysis on the CAS 200 were performed in the Molecular Pathology Laboratory of Columbia University Medical Center in New York City, according to standard protocols and institutional criteria [8,13].

### DNA index determination by ACIS

DNA content analysis on ACIS was performed according to the procedures suggested by the manufacturer. Briefly, the sections were cut at 7 μm and stained with Feulgen stain (ACIS kit), which includes hydrolysis with hydrochloric acid that removes non-nuclear substances and purines from the deoxyribose backbone of DNA molecules, followed by reaction with Schiff's reagent, an aqueous solution of cresyl-violet and sulfurous acid. Thus, the amount of Feulgen stain is directly proportional to the amount of DNA present in the nucleus. Feulgen-stained slides were automatically scanned with an Olympus microscope equipped with 3 Sony digital CCD chips at a speed of 30-frames/second. At a 40× magnification, the final estimated resolution was 250,000 per pixel (770,000 per airy disk), each individual Field of View (FOV) measured 160 × 120 μm, and there were157 × 209 (32,813) FOVs on a slide. Only the properly and uniformly stained slides were included. The final display was a 24-bit resolution (1280 × 1024), with the area of interest captured at 40× magnification. A daily quality control run with a standard calibration kit from the manufacturer was performed to ensure proper focus, black and white level balance for each microscope objective, and linear camera output.

The ACIS uses an inbuilt system that sets the filter threshold controlling the inclusion of stained nuclei at predefined (1–5) levels. The operator selects the level that helps the user separate/cut in between adjacent cells and cannot be identified by the software as individual cells, ranging from the least (level 1) to the most (level 5) aggressive. The system 'remembers' analysis regions to prevent the user from re-collecting nuclei from the same region. Unlike the CAS 200, no tissue correction factor is applied in the ACIS system to correct for underestimation of DI. To avoid inclusion of touching and overlapping nuclei, ACIS uses a set of image processing algorithms known as Watershed Segmentation. In these algorithms, nuclei that touch or overlap other nuclei are recognized through their size and other morphometric parameters and are separated by insertion of a single pixel-wide boundary at the point of contact. The analysis software provides five distinct cell separation profiles to allow optimal separation across a range of specimen morphologies, a process that works well for nuclei that touch or overlap to a modest degree. To avoid inclusion of excessively overlapped nuclei, each of the cell separation profiles was designed to recognize and exclude nuclei that exhibit excessive overlapping based on signature combinations of size, shape, color and morphometric filter descriptors.

**Figure 1:**
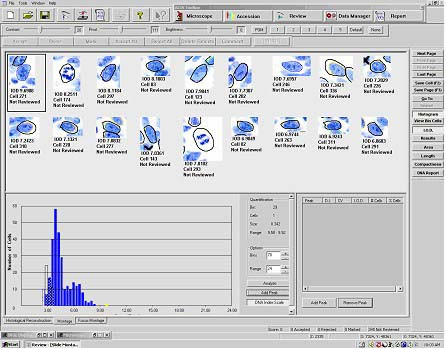
ACIS screen showing montage of microscopically selected and operator edited Feulgen-stained nuclei and the DNA content histogram generated on the basis of integrated optical density.

Overlapping nuclei, nuclear debris and other artifacts that escaped auto-detection and removal by the system were deleted by the operator. Qualified nuclei of approximately 30 control stromal cells, such as endothelial cells, macrophages, and fibroblasts, in the same tissue and about 200-targeted epithelial cells are obtained. DNA content histograms are automatically plotted in another window using the ACIS DNA ploidy software (Figure 1). The user can easily navigate between individual nuclei and their exact position on the DI histogram.

The mean integrated optical density (IOD) of control cells is assigned a DI of 1, which serves as an internal diploid (2N) standard and reference for DI calculation of the targeted cells. The IOD of control cells has a coefficient of variation (CV) of less than 10%. The histograms of the target cells showed a primary G0/G1 peak, and additional cells with or without well defined peaks with DI outside the G0/G1 peak (e.g., in S- or G2 phase of the cell cycle).

### Reproducibility of the ACIS

We randomly selected 30 samples of Barrett's lesions, including 5 cases each of gastric cardiac mucosa, SIM, ID (Indefinite for Dysplasia), LGD, HGD and EAC. To determine inter-observer variability, two trained, independent observers who were blinded to the diagnosis separately performed DI analysis on the same slide. To determine intra-observer reproducibility, the same observer performed DI analyses on the same slide at an interval of more than 15 days.

### Aneuploidy determination

Aneuploidy was diagnosed when the targeted epithelial nuclei showed no diploid G0/G1 peak and the DI of the G0/G1 peak was clearly positioned outside the diploid range.

### Statistical analysis

Statistical analysis was performed largely with Microsoft Excel. For linear regression analysis, the software SPSS (Chicago) was used. A *p *value of < 0.05 was considered statistically significant.

## Results

### DI values determined by the CAS 200 and ACIS systems

**Figure 2:**
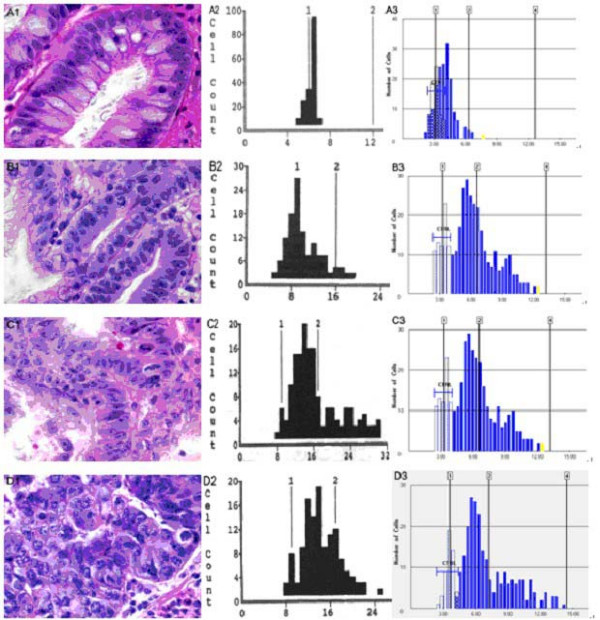
DI histograms generated by the CAS 200 (the middle column) and ACIS (the right column). The left column shows typical histology of the Barrett's related lesions (H&E stained, 400×) including specialized intestinal metaplasia without dysplasia (row A), low-grade dysplasia (row B), high-grade dysplasia (row C), and adenocarcinoma (row D). The corresponding DI histograms showed progressive increases in DNA content as disease progressed. On each DI histogram, the Y axis represents the number of total cells included and the X axis shows changes in DNA content in arbitrary units.

**Figure 3:**
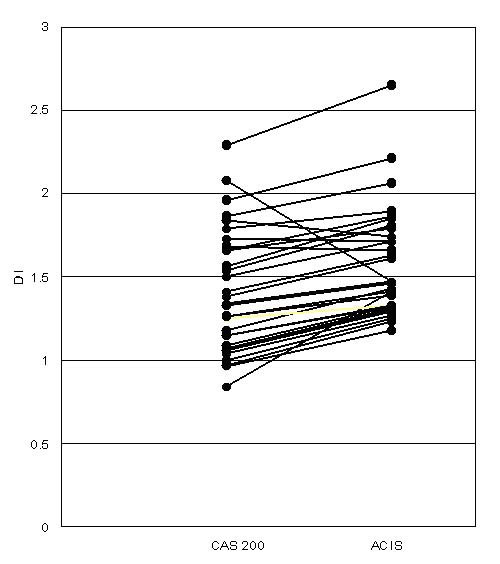
Comparison of DI values as determined by the CAS 200 and ACIS methods in various Barrett's lesions (*p *< 0.001, paired *t *test). Dots represent individual sections examined by both instruments.

**Figure 4:**
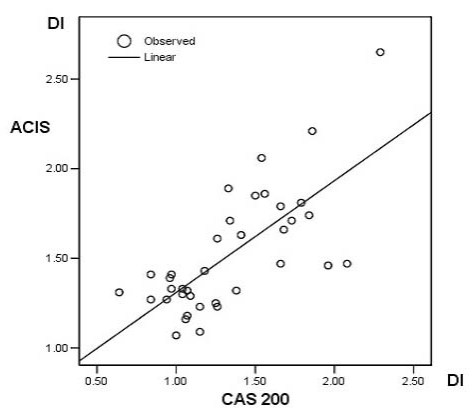
Linear regression analysis of DI values determined on the CAS 200 and ACIS instruments (n = 37). Note the excellent correlation obtained by the two systems. *R *= 0.89, *p *< 0.01 (two tailed).

Figure 2 shows examples of DI histograms obtained with the CAS 200 and ACIS instruments on distal esophageal tissue samples with various pathologic lesions. In general, we found that the ACIS yielded G0/G1 peaks with higher DI values than the CAS 200 system. When we assessed individual DI values of G0/G1 peaks obtained with the two systems, we again found that, in most cases and in all histopathological types of Barrett related lesions, the ACIS yielded higher DI values (*p* < 0.001, paired *t *test; Figure 3). On average, the DI values obtained with the ACIS were 18% higher than those obtained with the CAS 200. Both linear and logistical correlation analyses showed a statistically significant correlation between DI values generated by the two instruments (*p *< 0.01, two tailed; Figure 4).

### Ploidy analysis

Diagnoses of aneuploidy have been based on the upper limit of normal G0/G1 peak DI values, which vary from 1.1 to 1.29. Using 1.1 as the upper limit of the normal euploid range, CAS 200 diagnosed aneuploidy in 2/4 (50%) cases of SIM with no dysplasia, 3/7 (43%) cases of LGD, 11/12 (92%) cases of HGD, and 7/8 (88%) cases of EAC, whereas the ACIS diagnosed aneuploidy in all of these tissue samples (Table 1). Using 1.29 as the upper limit of the normal DI range, CAS 200 detected aneuploidy in 1/4 (25%) cases of SIM with no dysplasia, 0/7 (0%) cases of LGD, 10/12 (83%) cases of HGD, and 6/8 (75%) cases of EAC. In contrast, the ACIS diagnosed aneuploidy in 3/4 (75%) cases of SIM with no dysplasia, 4/7 (57%) cases of LGD, 11/12 (92%) cases of HGD, and 8/8 (100%) cases of EAC. Together, these findings indicate that the ACIS is more sensitive than the CAS 200 in diagnosing aneuploidy based on DI values of the G0/G1 peak.

**Table 1:** Prevalence of aneuploidy in various Barrett's lesions on the basis of 2 different DI cut-off upper limits

DI	≥1.1		≥1.3	
		
	CAS 200	ACIS	CAS 200	ACIS
C-GI	0/3 (0%)	0/3 (0%)	0/3 (0%)	0/3 (0%)
SIM	2/4(50%)	4/4(100%)	1/4(25%)	3/4(75%)
LGD	3/7(43%)	7/7(100%)	0/7(0%)	4/7(57%)
HGD	11/12(92%)	12/12(100%)	10/12(83%)	11/12(92%)
EAC	7/8(88%)	8/8(100%)	6/8(75%)	8/8(100%)

Total	23/34(68%)	31/34(91%)	17/34(50%)	26/34(76%)

Abbreviations: C-GI, normal gastrointestinal mucosa; SIM, specialized intestinal metaplasia; LGD, low grade dysplasia; HGD, high grade dysplasia; EAC, esophageal adenocarcinoma.

### Reproducibility of ACIS DI values

The DI values of G0/G1 peaks from an assortment of 30 Barrett's associated lesions were analyzed for inter- and intra-observer variability. For inter-observer variability, the Pearson coefficient of correlation was 0.917 (*p* < 0.0001, 2-tailed, n = 30), whereas, for intra-observer variability, it was 0.920 (*p* < 0.0001, 2-tailed, n = 30), thus showing that ACIS gives highly reproducible DI values.

## Discussion

Results have indicated that analysis of DNA ploidy may be a better method for the detection of pre-malignant lesions at high risk of malignant progression than histopathological dysplasia [4,8-10,14]. In addition, DNA aneuploidy may indicate the aggressiveness of malignant neoplasms in a variety of organs, including the prostate, breast, urothelial tract, cervix, ovary, lung, skin, and oral mucosa [3,4,15-20]. Moreover, very early DNA alterations, such as single chromosome or locus specific chromosomal abnormalities, may occur prior to expression of DNA abnormalities.

DNA ploidy analysis of solid tumors can be performed on cell suspensions using flow or image cytometry. Image cytometry performed on histological sections allows selective examination of the targeted epithelial cells. In addition, flow cytometry is less sensitive than image cytometry, and many lesions diagnosed as euploid on flow cytometry have been found to be aneuploid on image cytometry [6,7]. In Barrett's related esophageal lesions, however, only flow cytometry has been used in the diagnosis of large series of patients (14), indicating the need for large-scale studies of image cytometry in these lesions [8].

Diagnosis of aneuploidy by image cytometry has not been standardized. Different laboratories use different image cytometry systems, DNA staining techniques, section thicknesses, and types of control cells. Among the systems used are the CAS 100 [21] and CAS 200-Cell Analysis System Image Analyzers (Becton-Dickinson, San Jose, CA) [8,14,22,23], the QPATH (LEICA, Cambridge, England) [24], the MPV3 (Leitz, Wetzlar, Germany) equipped with a DNA cytometry system (ACAS, Ahrens, Bargteheide, Germany) [25], the Cyto-Savant Image Analyzer (Oncometrics Inc, British Columbia, Canada) [26,27]; the SAMBA 4000 Image Analyzer (Imaging Products International, Chantilly, VA) [28], and the Firefield ploidy system (Firefield Imaging Ltd, Nottingham, UK) [4,16]. The performance of these instruments has not been compared systematically and there is little published data comparing them. We therefore compared the results of DI analysis obtained by the ACIS and CAS 200 systems. We found that DI determination obtained by these two systems were well correlated, but that the DI values obtained with the ACIS were consistently higher than those acquired on the CAS 200. We also found that the ACIS was more sensitive in diagnosing aneuploidy, and that the protocol using the ACIS took less time than the CAS 200. Finally, the DI values obtained by the ACIS were highly reproducible.

We were somewhat surprised that the DI values obtained using the ACIS were consistently higher than those obtained using the CAS 200, inasmuch as similar image analysis systems should yield similar results. There are several possible explanations for this difference. First, the ChromaVision Blue Feulgen Stain Kit we used with the ACIS is composed of a Schiff reagent, which is truly stoichiometric, enabling a complete and thorough penetration of the stain and permitting uniform staining of the DNA molecules. Second, DI values are also dependent upon nuclear morphological features, including nuclear area, shape and density, and the ACIS system may more thoroughly account for these morphological features. Third, the tissue sections used for the ACIS (7 μm) were thicker than those used for the CAS 200 (5 μm). The latter system uses thinner sections to decrease the incidence of nuclear overlap, but it also yields an increased frequency of cut nuclei. The CAS 200 addressed this issue by using a tissue correction factor, which in essence is a right shift applied to the DI histogram and bringing the values into an acceptable numerical range. This correction may permit the detection of small aneuploid and tetraploid peaks, thereby preventing a 10% to 15% underestimate of aneuploid cases as diploid. The software, however, may not fully correct for nuclear truncation. In contrast, the thicker sections used in the ACIS markedly decrease the frequency of cut nuclei, thus avoiding the need for an artificial mathematical shift in the DI histograms. The ACIS uses a set of image processing algorithms (known as Watershed Segmentation) to consistently separate touching or moderately overlapping nuclei. In these algorithms, nuclei that touch or overlap are recognized through their size and other morphometric parameters, and are separated through the segmentation process by insertion of a single pixel-wide boundary between them at the point of contact. The analysis software provides five distinct cell separation profiles to allow optimal separation across a range of specimen morphologies. Each of these cell separation profiles is designed to recognize and exclude nuclei that exhibit excessive overlap based on signature combinations of size, shape, color and morphometric filter descriptors. In addition, the system allows the user to further delete any overlapping nuclear images and artifacts that may evade aneuploidy detection by ACIS. In this fashion, sufficient numbers of nuclei can be obtained from thicker sections without the complications of cut cells and the requirement for tissue correction factors.

Another difference between these two systems is in their control cells. The ACIS uses benign stromal cells, such as endothelial cells, macrophages, fibroblasts, and large lymphocytes, whereas the CAS 200 uses inactive small lymphocytes. Inactive small lymphocytes have more condensed nuclear chromatin structure than benign stromal cells and may not be stained optimally with the Feulgen dye. However, the lower IODs of lymphocytes would yield higher DI values for the epithelial cells. Some users of CAS 200 have also used external reference cells, such as rat hepatocytes or normal urothelial cells or human cerebellar cells. In general, selection of control cells may not make a dramatic difference in DI determination unless the controls are actively dividing cells such as gastrointestinal epithelial cells. It is therefore unlikely that the selection of control cells can explain the significant differences in the DI values generated by these two systems.

One of the main limitations of this study is that the two imaging systems were operated by different individuals, and operator related differences may yield different results. For example, differences in determining the threshold of digitized nuclear images may yield different values for DNA content, thereby changing the appearance of the DNA histograms. Moreover, the exact same cells were not analyzed by the two systems, adding to the variability in the results, although this difference was unlikely to produce consistently lower valves for the CAS 200.

The diagnosis of an aneuploid G0/G1 peak depends on its distinct separation from the normal diploid peak. Several performance measures have been proposed to allow image cytometry systems to identify aneuploidy if the DI peak deviates more than 10% from the diploid peak [29], but these measures are rarely reported in published studies [8,22-27]. Similarly, these studies rarely state the criteria by which the DI peak is determined. Euploidy (diploidy) may be diagnosed when cells in the G0/G1 peak have a mean DI value of less than 1.1 [22,23,26,27] or less than 1.3 [8,24,25]. In formalin-fixed gastrointestinal tissues, where the normal mucosa may have a mean DI value in the G0/G1 peak up to 1.29, a DI cut-off value of 1.3 seems more appropriate. In this study, we analyzed the prevalence of aneuploidy with the two cytometers using DI cut-off values of 1.1 and 1.30. We found that the ACIS was more sensitive than the CAS 200 in diagnosing aneuploidy using either cut-off value.

Aneuploidy has also been diagnosed in the presence of a normal euploid G0/G1 peak when additional aneuploid peaks, as evidenced by peaks of cells with DI values greater than those of the cells in the G0/G1 peak, are identified. However, what constitutes the aneuploid peaks is not clear. The number of nuclei in the G2 phase that are considered abnormal (tetraploid aneuploidy) varies from >10% [4] to >15% [8,27] to >25% [23]. Similarly, aneuploidy has been diagnosed when there are additional well-defined aneuploid peaks or when the number of nuclei with DI values in the aneuploid region constitute >1% of the total cells [4]. In this study, we did not diagnose aneuploidy when a normal diploid G0/G1 peak was present. Rather, we diagnosed aneuploidy only when the G0/G1 peak was clearly outside the diploid range. Since ACIS is more sensitive and yields higher DI values throughout the entire range of the DI, it will detect aneuploidy more frequently using similar diagnostic criteria.

To compare the two systems, we used an assortment of tissues with different histological types, including specialized intestinal metaplasia, various grades of dysplasia and adenocarcinoma, so that the full spectrum of pathological lesions may be covered. We found that the type of histology did not affect the results, in that the ACIS yielded higher DI values than the CAS 200 regardless of the type of histological lesions.

## Conclusion

ACIS provides a sensitive, efficient and highly reproducible method of DNA ploidy determination, and results obtained using this system correlated well with those obtained by the CAS 200. The ACIS system yielded DI values that were consistently higher than those obtained by the CAS 200. Variables such as the DNA stain used, the thickness of tissue sections, the tissue correction factor and the capture of morphological features of the nuclei may account for differences in results obtained with these two image cytometers. Moreover, automated image capture, semi-automated collection of cell nuclei, easy on-screen navigation and overall improved workflow may contribute to the shorter time needed for ploidy determination using the ACIS. Our findings indicate the importance of standardizing DNA content and ploidy determination methods.

## List of abbreviations used

ACIS, automated cellular imaging system

DI, DNA index

IOD, integrated optical density

CV, coefficient of variation

BE, Barrett's esophagus

SIM, specialized intestinal metaplasia

LGD, low-grade dysplasia

HGD, high-grade dysplasia

EAC, esophageal adenocarcinoma

## Competing interests

The author(s) declare that they have no competing interests.

## Authors' contributions

QH designed the project, participated in the data collection, and prepared the manuscript.

CY carried out the image analysis on ACIS, performed statistical analyses, and helped prepare the manuscript.

MF participated in the DNA ploidy analysis on CAS200.

MK participated in the histopathologic analysis of the specimens and edited the manuscript.

RG participated in the study design, statistical analysis, and manuscript preparation

## Pre-publication history

The pre-publication history for this paper can be accessed here:


